# Vasoactive peptides as biomarkers for the prediction of retinopathy of prematurity

**DOI:** 10.1038/s41390-024-03091-w

**Published:** 2024-02-24

**Authors:** Roland P. Neumann, Roland Gerull, Pascal W. Hasler, Sven Wellmann, Sven M. Schulzke

**Affiliations:** 1 Department of Neonatology, University Children’s Hospital Basel UKBB, University of Basel, Basel, Switzerland; 2 Division of Neonatology, University Children’s Hospital Inselspital Berne, Berne, Switzerland; 3 Department of Ophthalmology, University Hospital Basel, Basel, Switzerland; 4 Department of Neonatology, University Children’s Hospital Regensburg (KUNO), Hospital St. Hedwig of the Order of St. John, University of Regensburg, Regensburg, Germany

## Abstract

**Background:**

Retinopathy of prematurity (ROP) is a major complication in preterm infants. We assessed if plasma levels of midregional pro-atrial natriuretic peptide (MR-proANP) and C-terminal pro-endothelin-1 (CT-proET1) serve as early markers for subsequent ROP development in preterm infants <32 weeks gestation.

**Methods:**

Prospective, two-centre, observational cohort study. MR-proANP and CT-proET1 were measured on day seven of life. Associations with ROP ≥ stage II were investigated by univariable and multivariable logistic regression models.

**Results:**

We included 224 infants born at median (IQR) 29.6 (27.1–30.8) weeks gestation and birth weight of 1160 (860–1435) g. Nineteen patients developed ROP ≥ stage II. MR-proANP and CT-proET1 levels were higher in these infants (median (IQR) 864 (659–1564) pmol/L and 348 (300–382) pmol/L, respectively) compared to infants without ROP (median (IQR) 299 (210–502) pmol/L and 196 (156–268) pmol/L, respectively; both *P* < 0.001). MR-proANP and CT-proET1 levels were significantly associated with ROP ≥ stage II in univariable logistic regression models and after adjusting for co-factors, including gestational age and birth weight z-score.

**Conclusions:**

MR-proANP and CT-proET1 measured on day seven of life are strongly associated with ROP ≥ stage II in very preterm infants and might improve early prediction of ROP in the future.

**Impact:**

- Plasma levels of midregional pro-atrial natriuretic peptide and C-terminal pro-endothelin-1 measured on day seven of life in very preterm infants show a strong association with development of retinopathy of prematurity ≥ stage II.
- Both biomarkers have the potential to improve early prediction of retinopathy of prematurity.
- Vasoactive peptides might allow to reduce the proportion of screened infants substantially.

## Introduction

Retinopathy of prematurity (ROP) is a vascular proliferative disorder of the developing retina occurring in preterm infants. Whereas mild forms of ROP can resolve with few sequelae, severe ROP may result in irreversible visual impairment if not detected by ophthalmologic screening and treated timely.^1^ However, ophthalmologic examinations in preterm infants are demanding to perform and require a high level of training and expertise. ROP screening services are complex to organise with insufficient numbers of qualified ophthalmologists in certain areas of the world.^2^ Eye examinations are stressful and painful for affected infants. Furthermore, adverse events of ROP screening may occur, including apnoea, bradycardia, hypoxia, tachycardia, and emesis.^3^

In a previous study, we calculated the incidence of ROP in a population-based analysis of infants born below 32 weeks gestational age (GA) in Switzerland.^4^ We found that only 1.2% of these infants were diagnosed with ROP requiring treatment. In other words, the screening procedure had no therapeutic consequences for 98.8% of screened infants, questioning the currently installed screening criteria.^4^ New prediction models for ROP including biomarkers, demographic, and physiological parameters are under development.^5–7^ However, to date neither biomarker-based nor clinical prediction models are universally accepted to better define screening criteria for ROP in preterm infants. The main goal of any such effort is to reliably detect all cases of ROP requiring treatment while safely reducing the burden of screening in those who are unlikely to develop the disease.

Natriuretic peptides and corresponding natriuretic peptide receptors are present in the human retina.^8^ However, the role of natriuretic peptides in the development of ROP remains unclear. Previous studies suggest that urinary N-terminal B-type natriuretic peptide (BNP) prohormone levels measured on days 14 and 28 of life in preterm infants born below 30 weeks GA are strongly associated with the development of ROP requiring treatment^5^. Midregional pro-atrial natriuretic peptide (MR-proANP) is the mid-regional epitope of the ANP prohormone with a longer circulating half-life than mature ANP and therefore more suitable for analysis.^9^

Endothelin-1 (ET1) is a peptide hormone secreted by endothelial cells with strong vasoconstrictive properties. It is released in response to shear stress to endothelial cells, hypoxia, and hormones, including epinephrine and angiotensin-II. Its release is reduced by cyclic guanosine monophosphate, nitric oxide, and ANP.^10^ The endothelin system is activated in models of ischaemic retinopathy and mediates pathological vascular growth.^11^ However, the involvement of ET1 in the pathogenesis of ROP in preterm infants and its predictive ability towards ROP remains largely unknown. Whereas ET1 is difficult to measure due to its short half-life, the precursor peptide C-terminal proendothelin-1 (CT-proET1) is far more stable and available for estimation of ET1 levels.^12^ As both ANP and ET1 are involved in retinal growth and angiogenesis, we intended to explore the role of their stable biomarkers for early prediction of subsequent development of ROP.

We hypothesised that levels of MR-proANP and CT-proET1 are associated with subsequent development of ROP in very preterm infants. Specifically, we aimed to assess if plasma levels of MR-proANP and CT-proET1 on day seven of life are associated with the diagnosis of ROP ≥ stage II after adjusting for relevant demographic and clinical co-factors.

## Methods

### Study design

We conducted a prospective observational study in two tertiary care perinatal centres at the University Children’s Hospital Basel and the University Children’s Hospital Berne, both in Switzerland. Preterm infants with a GA below 32 weeks were eligible for inclusion in the study. Exclusion criteria were major congenital malformations or lack of parental consent. The Ethics Committee of Northwestern and Central Switzerland (Basel, study No. 233/13) and the Cantonal Ethics Committee (Berne, study No. 2481) approved the study. Written informed parental consent was obtained before enrolment. While the initial study prospectively assessed the association of biomarkers and respiratory morbidity^13^, the current study represents a post-hoc analysis to explore the association of MR-proANP and CT-proET1 with development of ROP. The blood sample collection was performed between November 2013 and November 2017.

### Biomarker analysis

Blood samples were taken in 500 μL EDTA tubes on day seven of life (±2 days) simultaneously with routine blood sampling. The samples were immediately centrifuged and stored at −80 °C. Analysis of MR-proANP and CT-proET1 was performed in three batches from 2015 to 2018 using an automated immunofluorescent assay (BRAHMS KRYPTOR, BRAHMS Biomarkers, Thermo Scientific, Henningsdorf, Germany).^9,12^ The functional assay sensitivities (20% coefficient of variation) were lower than 10 pmol/L for MR-proANP and 9.78 pmol/L for CT-proET1. Analytical detection limits were 2.1 pmol/L for MR-proANP and 2.94 pmol/L for CT-proET1.

### Ophthalmologic examinations

In our institutions, ROP screening was performed routinely in all infants born below 32 weeks GA and/or 1500 g birth weight. Experienced ophthalmologists examined the infants from a chronological age of 4 weeks but no earlier than 31 weeks postmenstrual age based on local standard screening protocols. Examinations were repeated in weekly or fortnightly intervals until complete vascularisation of the retina was observed. Retinal findings were classified according to the International Classification of Retinopathy of Prematurity (ICROP2) at the time of examination.^14^ In each participant, we recorded the most severe stage of ROP as documented by the ophthalmologists.

### Statistical analyses

Results for continuous variables are reported as median and interquartile range (IQR), and categorical variables are expressed as absolute frequency with corresponding percentage. Group comparison was performed by Wilcoxon–Mann–Whitney test, Kruskal Wallis test, and Chi-squared test as appropriate. We assessed the association of plasma levels of biomarkers (MR-proANP and CT-proET1) and demographic and clinical variables with (a) the occurrence of ROP of any stage and (b) with ROP ≥ stage II using univariable and multivariable logistic regression analyses. In univariable analyses, we considered the following explanatory variables: Biomarker levels (MR-proANP and CT-proET1), demographic factors known to influence outcomes of preterm infants (GA, birth weight, birth weight z-score, sex), and clinical characteristics including premature rupture of membranes, clinical chorioamnionitis, preeclampsia, markers of respiratory morbidity (surfactant administration, mechanical ventilation on day seven of life, need of supplemental oxygen on day seven of life, duration of supplemental oxygen), sepsis (defined as any episode of clinical and laboratory features compatible with sepsis with or without positive blood culture result), intraventricular haemorrhage (any stage), necrotising enterocolitis (Bell stage ≥ IIa), haemodynamically significant patent ductus arteriosus on day seven of life (hsPDA), and centre. For multivariable logistic regression models, we considered factors potentially influencing the outcome variable as identified in univariable regression analysis (*P* < 0.10). To avoid collinearity, birth weight z-score but not birth weight was added to models that included GA. Factors were explored using a stepwise backward elimination strategy, in which *P* value < 0.05 was considered statistically significant. We compared the predictive value of models using the likelihood-ratio test. The diagnostic utility of both biomarkers to discriminate between infants with and without subsequent development ROP ≥ stage II was investigated using receiver operating characteristic (ROC) curve analysis. Statistical analyses were performed using Stata 16.1 software (Stata Corporation, College Station, TX).

## Results

**Fig. 1:**
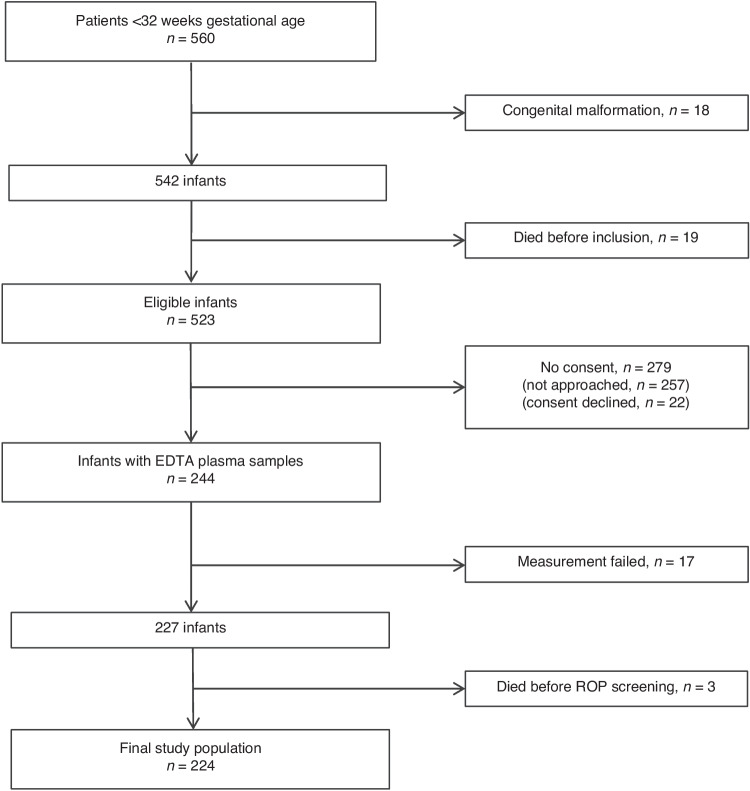
Flow chart of patient recruitment.

We prospectively recruited 244 preterm infants with a GA below 32 weeks during their first week of life for biomarker measurements. Biomarker measurement was not valid in 17 infants due to technical problems. Three infants died in the first weeks of life. Thus, we included 224 infants in the final dataset (Fig. 1). Biomarker levels and clinical characteristics of study participants are presented in Table 1. Median (IQR) GA was 29.6 (27.1–30.8) weeks, and median (IQR) birth weight was 1160 (860–1435) g. Nineteen infants (8.5%) were diagnosed with ROP ≥ stage II, seven of these (3.1%) developed ROP stage III, and two infants (0.9%) required laser therapy for ROP. As expected, infants with ROP had lower GA, birth weight, and birth weight z-score, and prolonged use of supplemental oxygen compared to those without ROP. The rate of sepsis and hsPDA on day seven of life was higher in infants with subsequent ROP development.

**Table 1:** MR-proANP and CT-proET1 levels and clinical characteristics of study population.

	No ROP (*n* = 199)	ROP stage I (*n* = 6)	ROP stage II (*n* = 12)	ROP stage III (*n* = 7)	ROP ≥ stage II (*n* = 19)
MR-proANP, pmol/L	299 (210–502)*	662 (397–778)*	788 (642–1487)*	905 (834–1641)*	864 (659–1564)
CT-proET1, pmol/L	196 (156–268)*	274 (255–371)*	346 (225–376)*	368 (300–407)*	348 (300–382)
Gestational age, weeks	29.9 (28.1–31.0)*	26.4 (25.6–27.9)*	25.5 (24.4–26.7)*	24.4 (24.1–24.7)*	24.7 (24.3–26.4)
Birth weight, g	1220 (920–1470)*	815 (660–980)*	742 (595–813)*	590 (540–670)*	650 (540–780)
Birth weight z-score	−0.1 (−0.7–0.3)**	−0.5 (−0.8 to −0.3)**	−0.7 (−1 to −0.1)**	−0.7 (−1.9 to −0.5)**	−0.7 (−1.3 to −0.2)
Male sex, *n* (%)	103 (52)	4 (67)	7 (58)	3 (43)	9 (47)
PROM, *n* (%)	56 (29)	1 (17)	4 (33)	0 (0)	4 (21)
Amnioninfection, *n* (%)	41 (21)	1 (17)	5 (42)	1 (14)	6 (32)
Preeclampsia, *n* (%)	28 (14)	1 (17)	3 (25)	0 (0)	3 (16)
Surfactant administration	97 (49)^‡^	4 (67)^‡^	11 (92)^‡^	6 (86)^‡^	17 (89)
Mechanical ventilation on DOL 7 (%)	10 (5)^†^	0 (0)^†^	3 (25)^†^	6 (86)^†^	9 (47)
Supplemental oxygen on DOL 7 (%)	26 (13)^†^	3 (50)^†^	5 (42)^†^	6 (86)^†^	11 (58)
Duration of supplemental O_2_, h	43 (1–390)*	660 (40–2198)*	1435 (559–1808)*	2616 (1686–2928)*	1693 (1128–2616)
Sepsis, *n* (%)	29 (15)^†^	2 (33)^†^	3 (25)^†^	6 (86)^†^	9 (47)
hsPDA on DOL 7, *n* (%)	35 (18)^†^	2 (33)^†^	6 (50)^†^	5 (71)^†^	11 (58)

Data are shown as median (IQR) unless stated otherwise.

*ROP* retinopathy of prematurity, *MR-proANP* midregional pro-atrial natriuretic peptide, *CT-proET1* C-terminal pro-endothelin-1, *PROM* premature rupture of membranes, *hsPDA* haemodynamically significant patent ductus arteriosus, *DOL* day of life.

*P value < 0.001, Kruskal-Wallis test; **P value = 0.030, Kruskal-Wallis test; ^†^*P* value < 0.001, Chi-squared test, ^‡^*P* = 0.007, Chi-squared test.

### Diagnostic utility of biomarkers

**Fig. 2:**
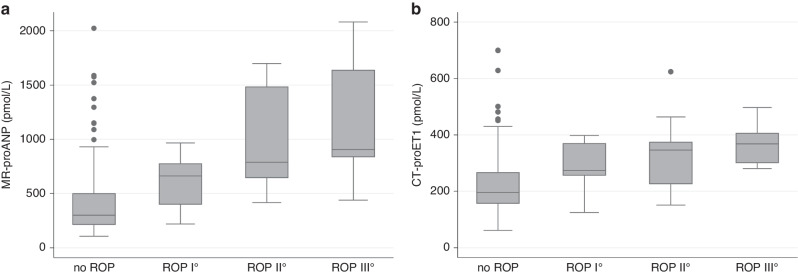
Levels of vasoactive peptides by ROP stages. **a** Levels of MR-proANP by ROP stages. **b** Levels of CT-proET1 by ROP stages.

MR-proANP levels on day seven of life were higher in infants with subsequent ROP compared to those without ROP. The levels were lowest in infants with no ROP (median (IQR) 299 (210–502) pmol/L) and increased gradually with severity of ROP up to a median (IQR) of 905 (834–1641) pmol/L in infants with ROP stage III. Similarly, levels of CT-proET1 on day seven of life were lowest in infants without subsequent ROP (median (IQR) 196 (156–268) pmol/L) and increased gradually with severity of ROP up to a median (IQR) of 368 (300–407) pmol/L in infants with ROP stage III (Table 1, Fig. 2).

To reach a sensitivity of 100% for detection of ROP ≥ stage II, we used MR-proANP with a cut-off of 400 pmol/L. This would have resulted in a specificity of 65.2% and in a reduction of screened infants to 40.6% (91/224 infants). For CT-proET1 we used 150 pmol/L as a cut-off aiming at a sensitivity of 100% for detection ROP ≥ stage II. This would have resulted in a specificity of 19.5%, corresponding to a modest reduction of screened infants to 82.1% (181/224 infants).

Combination of both biomarkers with the above-mentioned cut-off values would have allowed us to reduce the proportion of screened infants to 38.4% (86/224 infants), corresponding to a sensitivity of 100% and a specificity of 67.3% with a positive predictive value of 22.1% and a negative predictive value (NPV) of 100%.

### Results of logistic regression analyses

Univariable logistic regression analysis revealed that both biomarkers (MR-proANP and CT-proET1) showed a positive association with both any stage of ROP and ROP ≥ stage II. As expected, GA, birth weight and birth weight z-score were negatively associated with any stage of ROP and ROP ≥ stage II. Markers of respiratory morbidity (surfactant administration, mechanical ventilation on day 7 of life, need of supplemental oxygen on day 7 of life, duration of supplemental oxygen), sepsis, and hsPDA were positively associated with subsequent ROP development (Table 2).

**Table 2:** Results of univariable logistic regression analysis on any ROP and ROP ≥ stage II.

Explanatory variable	ROP any stage			ROP ≥ stage II		
	Coef *β*	95% CI	*P* value	Coef *β*	95% CI	*P* value
MR-proANP, pmol/L	0.003	0.002, 0.004	<0.001	0.003	0.002, 0.004	<0.001
CT-proET1, pmol/L	0.008	0.004, 0.011	<0.001	0.008	0.004, 0.012	<0.001
Gestational age, weeks	−0.756	−1.018, −0.494	<0.001	−0.826	−1.145, −0.508	<0.001
Birth weight, g	−0.006	−0.009, −0.004	<0.001	−0.007	−0.010, −0.003	<0.001
Birth weight z-score	−0.705	−1.205, −0.205	0.006	−0.729	−1.286, −0.171	0.010
Male sex	0.171	−0.666, 1.008	0.689	0.017	−0.923, 0.959	0.971
PROM	−0.492	−1.520, 0.536	0.349	−0.409	−1.554, 0.736	0.484
Amnioninfection	0.405	−0.533, 1.343	0.398	0.583	−0.442, 1.608	0.280
Preeclampsia	0.151	−0.990, 1.293	0.795	0.129	−1.165, 1.423	0.845
Surfactant administration	1.708	0.604, 2.813	0.002	2.169	0.678, 3.660	<0.001
Mechanical ventilation on DOL 7	2.181	1.178, 3.185	<0.001	2.683	1.610, 3.755	<0.001
Supplemental oxygen on DOL 7	2.104	1.217, 2.992	<0.001	2.093	1.104, 3.083	<0.001
Duration of supplemental oxygen, hours	0.002	0.001, 0.002	<0.001	0.002	0.001, 0.002	<0.001
Sepsis	1.527	0.644, 2.409	0.001	1.620	0.641, 2.600	0.002
Intraventricular haemorrhage	−0.357	−1.869, 1.154	0.643	−0.022	−1.552, 1.509	0.978
Necrotising enterocolitis	1.412	−1.021, 3.850	0.256	1.729	−0.719, 4.178	0.166
hsPDA on DOL 7	1.625	0.759, 2.490	<0.001	1.832	0.854, 2.809	<0.001
Centre	0.460	−0.383, 1.303	0.285	0.360	−0.596, 1.316	0.460

*ROP* retinopathy of prematurity, *Coef β* coefficient β, 95% *CI 95%* confidence interval, *MR-proANP* midregional pro-atrial natriuretic peptide, *CT-proET1* C-terminal pro-endothelin-1, *PROM* premature rupture of membranes, *DOL* day of life, *hsPDA* haemodynamically significant patent ductus arteriosus.

The association of MR-proANP with any stage of ROP persisted after adjusting for significant co-factors including GA and birth weight z-score. Multivariable analysis revealed an association of MR-proANP and ROP ≥ stage II after adjusting for GA (Table 3). The association of CT-proET1 with any stage of ROP and with ROP ≥ stage II persisted after adjusting for significant co-factors including GA and birth weight z-score (Table 4). In final models, markers of respiratory morbidity (surfactant administration, mechanical ventilation on day seven of life, need of supplemental oxygen on day seven of life, duration of supplemental oxygen), sepsis and hsPDA were no longer significantly associated with any stage of ROP or ROP ≥ stage II.

**Table 3:** Results of multivariable logistic regression analysis on any ROP and ROP ≥ stage II, including MR-proANP.

Explanatory variable	ROP any stage Pseudo *R*^2^ = 0.410, *P* < 0.001			ROP ≥ stage II Pseudo *R*^2^ = 0.427, *P* < 0.001		
	Coef *β*	95% CI	*P* value	Coef *β*	95% CI	*P* value
MR-proANP, pmol/L	0.001	0.000, 0.003	0.037	0.002	0.001, 0.003	0.003
Gestational age, weeks	−0.674	−0.975, −0.372	<0.001	−0.679	−1.032, −0.327	<0.001
Birth weight z-score	−0.724	−1.362, −0.086	0.026	ns	ns	ns

*ROP* retinopathy of prematurity, *MR-proANP* midregional pro-atrial natriuretic peptide, *Coef β* coefficient *β*, *95% CI* 95% confidence interval, *ns* non-significant.

**Table 4:** Results of multivariable logistic regression analysis on any ROP and ROP ≥ stage II, including CT-proET1.

Explanatory variable	ROP any stage Pseudo *R*^2^ = 0.387, *P* < 0.001			ROP ≥ stage II Pseudo *R*^2^ = 0.415, *P* < 0.001		
	Coef *β*	95% CI	*P* value	Coef *β*	95% CI	*P* value
CT-proET1, pmol/L	0.005	0.001, 0.009	0.044	0.005	0.001, 0.001	0.031
Gestational age, weeks	−0.706	−0.998, −0.413	<0.001	−0.733	−1.101, −0.397	<0.001
Birth weight z-score	−0.837	−1.494, −0.180	0.012	−0.807	−1.536, −0.078	0.030

*CT-proET1* C-terminal pro-endothelin-1, *Coef β* coefficient *β*, *95% CI* 95% confidence interval.

**Fig. 3:**
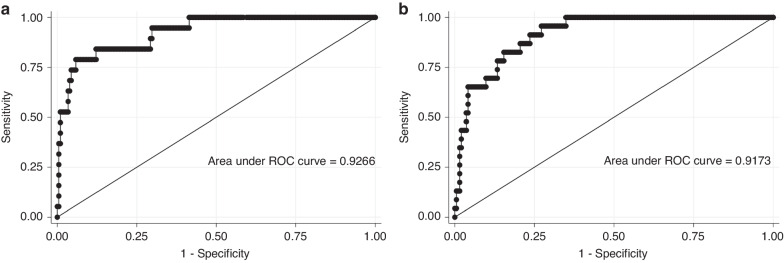
Receiver operating characteristic curves for prediction of ROP ≥ stage II in preterm infants born below 32 weeks of gestation. **a** Model including MR-proANP and gestational age. **b** Model including CT-proET1, gestational age, and birth weight z-score.

The area under the ROC curve (AUC) for subsequent development of ROP ≥ stage II for GA was 90.1. The final multivariable model, including MR-proANP and GA, improved the prognostic performance to 92.7. Similarly, the final multivariable model, including CT-proET1, GA, and birth weight z-score increased the AUC on ROP ≥ stage II to 91.7 (Fig. 3). Combination of both biomarkers did not increase prognostic performance.

## Discussion

Plasma MR-proANP and CT-proET1 levels on day seven of life in very preterm infants are strongly associated with the development of subsequent ROP after adjustment for relevant co-factors. Both biomarkers have the potential to improve ROP prediction models in the future and might help tailoring ROP screening protocols.

To the best of our knowledge, this is the first study investigating the association of MR-proANP and CT-proET1 with ROP in very preterm infants. Previous studies used BNP to estimate the risk of ROP: Czernik et al. studied the urinary NT-proBNP normalised to creatinine in 136 very preterm infants on days seven, 14, and 28 of life.^15^ They found a strong association of this biomarker with the development of severe ROP defined as stage III or stage II requiring surgery.^15^ To validate the findings in a larger population, Bührer et al. performed a prospective multinational study (*n* = 837) to assess the capacity of urinary NT-proBNP on days 14 and 28 of life for prediction of ROP requiring treatment in infants born below 30 weeks GA.^5^ They similarly found a strong association between urinary NT-proBNP and subsequent development of severe ROP. However, overlapping ranges of NT-proBNP of infants with and without subsequent ROP precluded the authors to propose a safe algorithm to reduce the number of eye examinations. Ding et al. retrospectively developed a ROP prediction model including serum NT-proBNP levels and clinical parameters.^16^ The best model (AUC 0.9) included GA, birth weight, weight gain rate, invasive mechanical ventilation and NT-proBNP. However, some of the variables in this model were collected up to the day of first ROP screening, thus, timely stratification of patients to reduce screening burden would not be possible using this approach.

Strengths of our study are that we prospectively collected a comprehensive dataset for evaluation of the two biomarkers along with key factors of neonatal morbidity across two centres, allowing us to include a considerable number of study participants, although the proportion of infants with ROP ≥ stage III and ROP requiring treatment was low. Limitations include a single time point of measurement of both biomarkers precluding assessment of longitudinal dynamics and the lack of a validation cohort.

ANP is secreted by cardiomyocytes in response to cardiac stretch of the atria.^17^ ANP can indicate left ventricular volume overload in children with heart failure.^18^ In very preterm infants, MR-proANP was associated with the presence of hsPDA.^19^ Also, Gerull et al. found that MR-proANP (as well as CT-proET1) serve as early markers of respiratory morbidity.^13^ In comparison, Endothelin-1 is released by endothelial cells in response to sheer stress and hypoxaemia. It is elevated in newborn infants with persistent pulmonary hypertension and in preterm infants with respiratory distress.^20,21^ Therefore, elevated levels of both MR-proANP and CT-proET1 may simply reflect increased cardiorespiratory morbidity in the most immature and smallest infants who also tend to have the highest risk for the development of ROP. Bührer et al. hypothesised that circulatory compromise itself may contribute to the development of ROP besides well-established clinical risk factors including low GA, oxygen exposure, hsPDA, sepsis and poor postnatal weight gain.^5^

To assess the influence of cardiorespiratory morbidity on ROP development, we explored several parameters of cardiorespiratory compromise (surfactant administration, mechanical ventilation on day seven of life, need of supplemental oxygen on day seven of life, duration of supplemental oxygen, sepsis, and hsPDA) in univariable and multivariable regression analyses. These factors were associated with the development of any stage ROP and ROP ≥ stage II in univariable analysis. However, multivariable analysis revealed only GA, birth weight z-score, MR-proANP, and CT-proET1 as independent risk factors. Markers of respiratory morbidity, sepsis, and interestingly not hsPDA, which itself is associated with MR-proANP and CT-proET1, were no longer associated with the development of any stage ROP and ROP ≥ stage II after adjustment for co-factors.^22^ This suggests that ANP and ET1 might have an intrinsic effect on retinal vascularisation and ROP development but involved mechanisms are not fully understood.

Natriuretic peptides, including ANP and BNP are not only synthesised in the heart but also in other body tissues, such as the human retina, although at lower levels.^8^ They play a role in retinal vascular growth and can influence release of vascular endothelial growth factor (VEGF).^23,24^ In a rodent model of choroidal neovascularisation, ANP reduces VEGF production, vascular leakage, and neovascularisation after laser-induced injury of the retina.^24^ Potentially, a pronounced suppression of VEGF by ANP directly after birth might influence phase 1 of ROP (vessel growth cessation) as well as phase 2 (retinal neovascularisation) in human preterm infants.

Patel et al. demonstrated the involvement of the endothelin system in pathological neovascularisation in an animal model of oxygen-induced retinopathy.^11^ Specifically, endothelin production was induced by hypoxia. Blockade of the endothelin receptor B resulted in decreased expression of VEGF and other angiogenic mediators, and in a reduction of pathological vascular development in the retina.^11^ Although we did not measure MR-proANP or CT-proET1 at the time when the ROP progressed in phase 2, it is conceivable that elevations of these two biomarkers at any time point of retinal development might disturb the delicate balance of vasoactive substances in the immature retina and promote the development of ROP.

Ophthalmologic examinations for ROP screening impose a high burden on patients, parents, and medical resources. We identified plasma MR-proANP and CT-proET1 as independent risk factors for the development of ROP. These readily available biomarkers have the potential to early identify infants at high risk of developing ROP≥ stage II who may require ROP treatment. Single use of MR-proANP or, for slightly improved utility, combined use of both biomarkers for ROP prediction on day seven of life would have allowed us to reduce the proportion of infants having ophthalmological examinations by about 60%, with a sensitivity of 100% for detection of ROP ≥ stage II.

Several promising ROP prediction models have been proposed, such as WINROP (based on GA, birth weight, postnatal weight gain as a surrogate of insulin-like growth factor IGF-1)^25^, CHOP ROP (based on birth weight, GA, and postnatal weight gain)^26^ or ROPScore (based on GA, birth weight, blood transfusions, mechanical ventilation, and postnatal weight gain)^27^. However, validation studies of these models in different populations revealed a lack of sensitivity.^28–30^ Arguably, including biomarkers such as MR-proANP and CT-proET1 in these established clinical ROP prediction models may improve their predictive value and their sensitivity.

Longitudinal measurements of plasma MR-proANP and CT-proET1 in preterm infants at risk for ROP are needed to determine the time point of highest predictive power for ROP development with 100% sensitivity and high specificity. Findings of our study need to be assessed longitudinally in large, multi-centre validation cohorts to confirm the utility of these biomarkers prior to inclusion in clinical practice. The performance of both biomarkers needs to be tested in combination with established models for the prediction of severe ROP.

In conclusion, plasma biomarkers MR-proANP and CT-proET1 were strongly associated with ROP development in very preterm infants. Both biomarkers may help to improve early prediction of ROP, optimise target population, and reduce the screening burden related to ROP.

## Data Availability

The datasets generated during and/or analysed during the current study are available from the corresponding author upon reasonable request.
